# Functional mining of novel terpene synthases from metagenomes

**DOI:** 10.1186/s13068-022-02189-9

**Published:** 2022-10-08

**Authors:** Suryang Kwak, Nathan Crook, Aki Yoneda, Naomi Ahn, Jie Ning, Jiye Cheng, Gautam Dantas

**Affiliations:** 1 The Edison Family Center for Genome Sciences & Systems Biology, Washington University School of Medicine in St. Louis, 4515 McKinley Avenue, Room 5121, Campus Box 8510, Saint Louis, MO 63110 USA; 2 Department of Pathology and Immunology, Washington University School of Medicine in St. Louis, Saint Louis, MO 63110 USA; 3 Department of Chemical and Biomolecular Engineering, North Carolina State University, Raleigh, NC 27695 USA; 4 Department of Biomedical Engineering, Washington University in St. Louis, Saint Louis, MO 63130 USA; 5 Department of Molecular Microbiology, Washington University School of Medicine in St. Louis, Saint Louis, MO 63110 USA

**Keywords:** Functional metagenomics, Terpene synthase, Prenyl pyrophosphate, β-Farnesene

## Abstract

**Background:**

Terpenes are one of the most diverse and abundant classes of natural biomolecules, collectively enabling a variety of therapeutic, energy, and cosmetic applications. Recent genomics investigations have predicted a large untapped reservoir of bacterial terpene synthases residing in the genomes of uncultivated organisms living in the soil, indicating a vast array of putative terpenoids waiting to be discovered.

**Results:**

We aimed to develop a high-throughput functional metagenomic screening system for identifying novel terpene synthases from bacterial metagenomes by relieving the toxicity of terpene biosynthesis precursors to the *Escherichia coli* host. The precursor toxicity was achieved using an inducible operon encoding the prenyl pyrophosphate synthetic pathway and supplementation of the mevalonate precursor. Host strain and screening procedures were finely optimized to minimize false positives arising from spontaneous mutations, which avoid the precursor toxicity. Our functional metagenomic screening of human fecal metagenomes yielded a novel β-farnesene synthase, which does not show amino acid sequence similarity to known β-farnesene synthases. Engineered *S. cerevisiae* expressing the screened β-farnesene synthase produced 120 mg/L β-farnesene from glucose (2.86 mg/g glucose) with a productivity of 0.721 g/L∙h.

**Conclusions:**

A unique functional metagenomic screening procedure was established for screening terpene synthases from metagenomic libraries. This research proves the potential of functional metagenomics as a sequence-independent avenue for isolating targeted enzymes from uncultivated organisms in various environmental habitats.

**Supplementary Information:**

The online version contains supplementary material available at 10.1186/s13068-022-02189-9.

## Background

Terpenes are the largest and structurally most diverse group of natural organic products [1]. Their functions in living organisms are essential and as diverse as their structures, ranging from common metabolites to highly specialized functional molecules [1, 2]. Terpenes and their derivatives are commercially valuable resources for flavors, cosmetics, medicines, and biofuels [3]. Terpenes have commonly been extracted from natural sources for commercial production, but the extraction processes are often limited by low concentrations in these settings [2]. Alternative chemical syntheses of terpenes are expensive and low-yielding due to the structural complexity of terpene molecules [2, 4]. Therefore, microbial engineering for overproducing terpenes from inexpensive sugars has become a scalable, cost-effective, and environmentally friendly alternative to the conventional approaches [2].

*Escherichia coli* and *Saccharomyces cerevisiae* are the two host systems that have been most actively engineered as platforms for the biosynthesis of terpenes and their derivatives [5]. Both strains have native metabolic pathways generating prenyl pyrophosphate precursors for the terpene biosynthesis, namely, the 2-C-methyl-D-erythritol 4-phosphate (MEP) pathway in *E. coli* and the mevalonate (MVA) pathway in *S. cerevisiae* [6]. Nevertheless, optimization of metabolic fluxes through the pathways is necessary to overproduce terpenes in those host systems [2]. In the case of *E. coli*, overexpression of key enzymes of the native MEP pathway or the heterogenous MVA pathway has successfully enhanced the production of terpenes and terpene derivatives [7–10]. However, excessive prenyl pyrophosphate pools resulting from the overexpression of key enzymes in the MEP and MVA pathways severely inhibit the growth of host *E. coli*; this precursor toxicity can be alleviated by channeling the excessive prenyl pyrophosphates into terpenes through an exogenous terpene synthase [9].

Identification of novel terpene synthases (TSs) can immensely expand the range of terpene products and their applications, together with rapid advances in platform microbial engineering [11–14]. However, compared to the high number of structurally characterized terpenes, the identification of terpene biosynthetic elements remains limited [15]. Thanks to rapidly growing genomic and metagenomic sequencing data, high-throughput prediction approaches based on amino acid sequence similarity have emerged to efficiently screen potential TS genes [16]. However, these approaches have been limited in the prediction of bacterial TSs because of their low levels of sequence similarity to plant and fungal TSs and their relatively low mutual similarities [17]. Previous studies mining TSs from bacterial genome databases using profile hidden Markov models (HMMs) demonstrated the promise of bacterial communities as bioresources of novel TSs [17–19].

In a complementary approach, Withers et al. showed the potential of function-based genome mining to screen biosynthetic elements of terpene and terpene derivatives from bacterial genomes. The system employed the toxicity of excessive prenyl pyrophosphates in *E. coli* as a selection pressure. Two isopentenol biosynthetic enzymes, which mitigate the growth inhibition by consuming the toxic precursors, were discovered from *Bacillus subtilis* genomic DNA libraries through this system [20]. Similarly, Furubayashi et al. developed another functional TS screening method based on the colorimetric changes of *E. coli* by active TSs that compete with carotenoid pathways for prenyl pyrophosphate precursors [21].

**Fig. 1:**
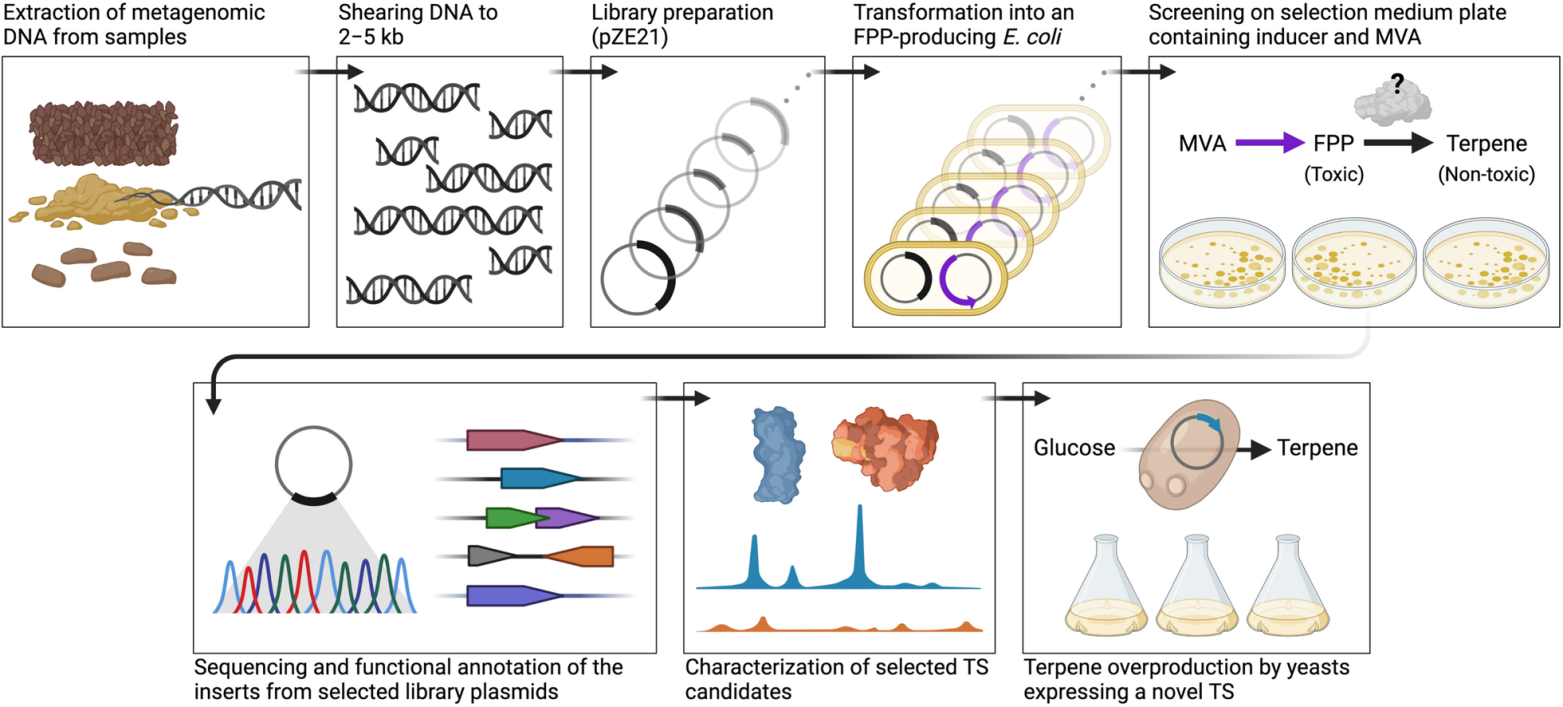
Schematic illustration of the precursor toxicity-based functional metagenomic screening system

Here we present a functional metagenomics approach for screening TSs by finely optimizing the precursor toxicity mechanism [9, 20] and expanding the range of novel TS sources from single microorganisms to bacterial metagenomes. Our approach achieved a unique and effective platform for isolating TS genes from bioresources independently of bacterial cultures or genome databases (Fig. 1). We demonstrated the potential of functional metagenomic screening by isolating a novel β-farnesene synthase from a human fecal metagenomic library that does not show amino acid sequence similarity to known β-farnesene synthases.

## Results and discussion

### Optimization of the selection pressure for the precursor toxicity-based screening

**Fig. 2:**
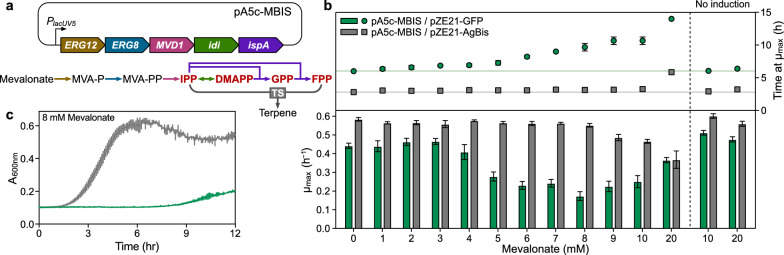
Construction and optimization of the precursor toxicity-based functional metagenomic screening system. **a** Structure of pA5c–MBIS plasmid overexpressing the prenyl pyrophosphate precursors synthetic (MBIS) pathway under the control of IPTG-inducible *P*_*lacUV5*_. A terpene synthase mitigates the toxicity of excessive prenyl pyrophosphate precursors (red) by converting them into non-toxic terpene molecules. **b** Gap between growth inhibitions on the negative (green) and positive (gray) controls was maximized by adjusting the concentration of supplemented mevalonate. The MBIS operon expression was induced by 0.5 mM IPTG. Upper panel, the timepoint the strain exhibited maximum specific growth rate (*μ*_max_) with selected mevalonate concentration. Lower panel, maximum specific growth rate. **c** Growth profiles of negative and positive controls in the optimized screening medium containing 8 mM mevalonate

We hypothesized that the toxicity of prenyl pyrophosphate pools, namely, precursors of terpene biosynthesis, could be harnessed to functionally search through the vastly uncharacterized genomic content of environmental microbial communities for enzymes catalyzing terpene biosynthesis. To test this hypothesis, we devised a precursor toxicity-based functional metagenomics approach (Fig. 1). We introduced the synthetic MBIS operon consisting of *S. cerevisiae ERG12*, *ERG8*, *MVD1*, and *E. coli idi* and *ispA* [9] under the control of an isopropylthio-β-galactoside (IPTG)-inducible promoter *P*_*lacUV5*_, to ensure the overproduction of prenyl pyrophosphates in *E. coli*. We used pA5c carrying chloramphenicol resistance and the p15A origin of replication as a backbone vector of this synthetic operon, considering its compatibility with pZE21-based metagenomic library plasmids containing the kanamycin resistance marker and pBR322 origin of replication. The resulting plasmid, pA5c–MBIS, enabled *E. coli* to biosynthesize prenyl pyrophosphates from supplemented mevalonate with IPTG induction (Fig. 2a). Two control plasmids were constructed to determine the optimal concentration of mevalonate as a selection pressure for isolating genes encoding TS. pZE21–AgBis carrying the bisabolene synthase gene from *Abies grandis* (AgBis), one of the best-characterized sesquiterpene synthases [10, 22], was constructed as a positive control. We substituted the green fluorescent protein (GFP) gene for AgBis to prepare a negative control plasmid pZE21–GFP. We tracked the growths of the two *E. coli* DH10B strains carrying pA5c–MBIS and one of the control plasmids on varied mevalonate concentrations in the medium from 0 to 20 mM, with or without induction of the MBIS operon. Mevalonate supplementation alone did not inhibit the growth of both control *E. coli* strains without induction (Fig. 2b). With induction, mevalonate hindered the growth of the negative control strain; mevalonate concentration was positively correlated with the time required for the negative control *E. coli* to reach the maximum specific growth rate (Fig. 2b and Additional file 1: Fig. S1). On the other hand, the growth inhibition by mevalonate was substantially alleviated in the positive control *E. coli* (Fig. 2b and Additional file 1: Fig. S1), corroborating the conversion of the excess prenyl pyrophosphate pools to bisabolene that is not toxic to *E. coli* [10]. 8 mM of mevalonate efficiently inhibited the growth of negative control without damage to the fitness of positive control (Fig. 2b, c).

### Minimizing spontaneous mutations

Spontaneous mutations under lethal stresses may allow *E. coli* to evade the selection pressure of excessive prenyl pyrophosphates, obviating the need for TS activities from the library plasmid [23]. To minimize the spontaneous mutation rate during the precursor toxicity-based screening, we adopted a new *E. coli* host strain, LowMut, a reduced-genome strain lacking most genes irrelevant for laboratory applications, including active IS elements and error-prone DNA polymerases [24–26]. We compared the stabilities of the two host strains, DH10B and LowMut, on the screening plate after transformation with pA5c–MBIS and one of positive (pZE21–AgBis) and negative (pZE21–GFP) control plasmids. To avoid potential carry-over transmission of free mobile DNA elements, such as transposases, integrases, and recombinases [27] from the original cloning host into LowMut, plasmid transformation into LowMut, re-extraction, and re-transformation were repeated 3 times.

**Fig. 3:**
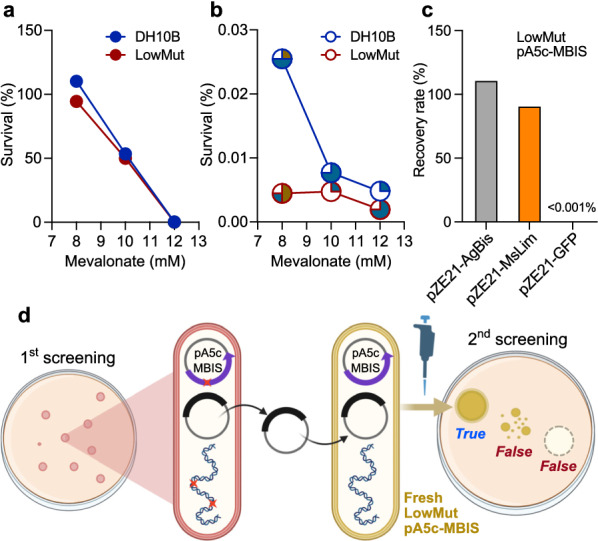
Control of spontaneous mutagenesis during the precursor toxicity-based screening. **a**, **b** Survival rates of DH10B- and LowMut-based positive **a** and negative **b** controls on the screening plate with 1 mM IPTG and a variation of mevalonate concentration. Survival rate was the ratio of colony forming units (CFUs) on the selected mevalonate concentration to CFUs on 0 mM mevalonate. Pie graphs in **b** represent the location of mutagenesis in the MBIS operon of 4 randomly selected colonies from each condition. White, no mutation in the MBIS operon; olive, ERG12; cyan, ERG8 (see the color scheme of Fig. 1a). **c** Recovery of LowMut-based positive control strains from a simulated mixture on the screening plate with 1 mM IPTG and 8 mM mevalonate. **d** Scheme of the second screening process using fresh LowMut pA5c–MBIS strain to exclude false positives that evade the precursor toxicity via spontaneous mutations (red crosses)

Positive controls of both host strains exhibited comparable colony numbers on the 8 mM mevalonate plate and the non-selective plate with no mevalonate (Fig. 3a). On the other hand, the negative control of LowMut exhibited a significantly lower colony number on the 8 mM mevalonate plate than that of DH10B (Fig. 3b), indicating that LowMut is a more suitable host than DH10B regarding genetic stability under the selection pressure of excessive prenyl pyrophosphates. Higher mevalonate concentrations than 8 mM diminished the number of colonies of both positive and negative control strains, regardless of the host system (Fig. 3a, b), similar to the trend observed in the liquid cultivations for optimizing the mevalonate concentration (Fig. 2b). We collected 4 colonies of the negative controls from each screening medium agar plate to check for potential mutations in the pA5c–MBIS plasmid responsible for generating prenyl pyrophosphates. Indeed, 14 among the 24 colonies contained transposon insertions that directly interrupted open reading frames (ORFs) of the MBIS operon in the pA5c–MBIS. All insertions were in the first (*ERG12*) or second (*ERG8*) ORFs, while the promoter *P*_*lacUV5*_ and following ORFs of the MBIS operon (*Mvd1*, *idi*, *ispA*) were not perturbed in any tested colonies (Fig. 3b). The other 10 colonies did not show any mutation on the MBIS pathway, suggesting that spontaneous mutations in the genomic DNA might rearrange the metabolism of *E. coli* to mitigate the toxicity of excessive prenyl pyrophosphates [23].

### Validation of the optimized precursor toxicity-based screening procedure

To validate the capability of the optimized toxicity-based screening to isolate TSs from libraries containing both TS ORFs and non-TS ORFs, the screening procedure was simulated with well-characterized TSs and GFP. We adopted limonene synthase from *Mentha spicata* (MsLim) as a model monoterpene synthase in addition to the sesquiterpene synthase AgBis. To construct MsLim-expressing positive control, pZE21–MsLim was transformed into the LowMut pA5c–MBIS after 3 transformation–extraction passages through LowMut, as described above. Cells of the two LowMut-based positive controls expressing one of the model terpene synthases were mixed with approximately 10^5^-fold more cells of LowMut-based negative control expressing GFP (Additional file 1: Fig. S2a, b). The toxicity-based screening on 1 mM IPTG and 8 mM mevalonate successfully recovered the positive control strains from the artificial library mixture (Fig. 3c and Additional file 1: Fig. S2). Still, a few colonies grown on screening plates were negative controls (Additional file 1: Fig. S2b, c), reaffirming that the LowMut host strain can overcome the designated selection pressure via other avenues than terpene synthase activity. The high recovery rate and decent selectivity validated in this simulation demonstrate the reliability of precursor toxicity-based screening as an avenue for isolating varied terpene synthases consuming farnesyl pyrophosphate (FPP) and geranyl pyrophosphate (GPP).

### Screening of potential TSs from metagenomic libraries

Human fecal and soil metagenomic library plasmids were transformed into the LowMut pA5c–MBIS strain (see “Minimizing spontaneous mutations” section for detailed description). The resulting library cells were incubated on solid media screening plates. For the first screening, growth patterns of the library transformants were compared with the positive (LowMut pA5c–MBIS pZE21–AgBis) and negative (LowMut pA5c–MBIS pZE21–GFP) control strains, and the colonies showing similar growth to the positive control were selected as transformants expressing a potential TS. We double-checked the first screening outcomes by retransforming the screened library plasmids into fresh LowMut pA5c–MBIS cells and spotting each transformant on the screening plate. The library plasmids whose new transformants did not show uniform growth were excluded as false positives; their original colonies from the first screening might overcome the precursor toxicity through spontaneous mutagenesis (Fig. 3d). It has been previously shown that mixed colony sizes may represent high mutator phenotypes allowing a fast response to the toxicity even during the second screening [23]. 27 library plasmids were screened through the precursor toxicity-based functional metagenomic screening (Additional file 1: Fig. S3). The insert regions of screened library plasmids were sequenced, putative ORFs were predicted from assembled contigs, and their amino acid sequences were further analyzed to predict their potential functions.

Despite these screening steps, we identified false positives from the selected libraries which apparently evaded the selection pressure without bona fide TS activity. 6 library plasmids included ORFs predicted to encode multidrug and toxic compound extrusion (MATE), auxin efflux carrier (AEC), or ATP-binding cassette (ABC) transporters (Additional file 1: Fig. S3) which may transport excessive intracellular prenyl pyrophosphates out from the *E. coli* cells, thereby alleviating intracellular toxicity. We also found 2 library plasmids expressing a transcriptional repressor and a putative transposase (Additional file 1: Fig. S3) which might induce fast spontaneous mutations inactivating a part of the MBIS operon or rearranging host metabolism. In addition, some of the selected library plasmids carried ORFs that are likely too short to be potential TSs (< 450 bp) [28] or did not have any ORFs (Additional file 1: Fig. S3), probably owing to the spontaneous mutagenesis (Fig. 3a, b). Finally, we screened 10 unique TS candidates from 15 screened library plasmids (Additional file 1: Fig. S3).

### Characterization of TS candidates

Amino acid sequences of all ORFs from the selected library plasmids were novel (see Additional file 1: Supplementary texts), except TS36R1, which has an identical amino acid sequence to a histidine phosphatase family protein identified from *Faecalibacterium prausnitzii* (NCBI Reference Sequence: WP_158395513.1). The other 9 novel proteins of potential TS activity were cloned into the pET28b( +) backbone for the functional validation of the selected candidates. However, the cloning of most selected ORFs was unsuccessful regardless of the location of the hexa histidine tag; their cloning yield was extremely low, and all the resulting plasmids either destroyed the start codon or frameshifted the insert. TS10F1 was the only protein whose ORF was successfully cloned into pET28b( +) among the 9 candidates.

**Fig. 4:**
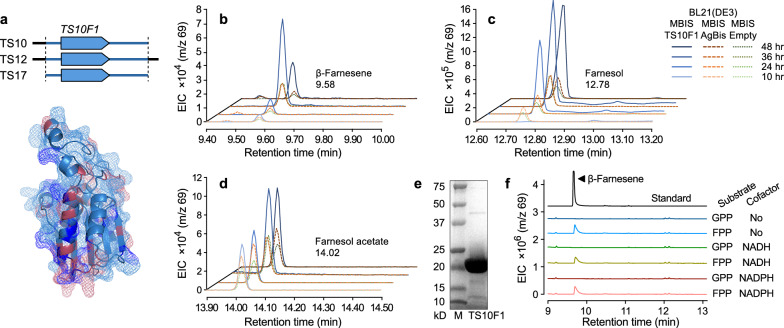
Characterization of TS10F1. **a** TS10F1 ORF was isolated from 3 different contigs screened from a human fecal metagenome library 40301 (Additional file 1: Fig. S2). TS10F1 showed 75% identity to a histidine phosphatase family protein of *Prevotella pectinovora* (sky blue, correct match; blue, functional equivalence; plum, not similar amino acid). **b** − **d** Gas chromatography–mass spectrometry (GC–MS) analysis of the TS10F1 in vivo test cultures. With IPTG induction of the MBIS pathway and supplemented mevalonate, BM_TS10F1 (blue) generated more β-farnesene and its derivatives than BM_AgBis (orange) and BM_Empty (green, no IPTG induction). **e** Confirmation after the purification of TS10F1 (estimated size is 21.54 KDa). M, protein marker. **f** GC–MS analysis of in vitro enzyme assay products at a 3 h timepoint and β-farnesene standard (top)

The contig containing TS10F1 was discovered from multiple library plasmids of a human fecal metagenome (Fig. 4a). TS10F1 exhibited 75% identity to potential histidine phosphatase family protein of *Prevotella pectinovora* (NCBI Reference Sequence: WP_044075392.1), and the predicted catalytic core regions of the two proteins were conserved (Fig. 4a and Additional file 1: Fig. S4). We tested in vivo functionality of TS10F1 by cultivating recombinant BL21(DE3) carrying both pA5c–MBIS and pET28–TS10F1 (BM_TS10F1) in a defined medium with a dodecane overlay to capture potential terpene products. Positive control BM_AgBis (pA5c–MBIS and pET28–AgBis) and negative control BM_Empty (pA5c–MBIS and pET28) were cultivated in the same culture condition to define BM_TS10F1-specific terpene-like molecules; IPTG was not added to the BM_Empty culture due to the precursor toxicity by pA5c–MBIS. BM_TS10F1 generated more β-farnesene and its derivatives, such as farnesol and farnesol acetate, than control strains (Fig. 4b, d).

The β-farnesene production in detectable levels by the positive and negative control strains connotes the evasion of the toxic accumulation of FPP by the plasticity of terpenoid biosynthesis in *E. coli* [9, 29]. In addition, innate *E. coli* phosphatases catalyzing farnesol formation from FPP, such as AphA, PgpA, and PgpB [30], might cause the basal farnesol levels of the two control strains. Furthermore, chloramphenicol acetyltransferase, the chloramphenicol resistance marker in the pA5c–MBIS plasmid, catalyzes nonspecific acetylation toward terpene alcohols, such as farnesol acetate formation [31]. We hypothesized that TS10F1 increased β-farnesene production through its β-farnesene synthase activity, and the native promiscuous *E. coli* metabolic activities and above-mentioned enzymes caused additional farnesol and farnesol acetate accumulation by hydrating and esterifying the excess β-farnesene [32]. To test this hypothesis, we performed an enzyme assay of the purified TS10F1 (Fig. 4e). Indeed, TS10F1 generated β-farnesene from FPP, not from GPP, regardless of cofactors (Fig. 4f). There was no spontaneous conversion of FPP into β-farnesene (Additional file 1: Fig. S5), suggesting that TS10F1 possesses the β-farnesene synthase activity removing diphosphate from FPP (EC 4.2.3.47).

### Farnesene overproduction by recombinant yeast expressing TS10F1

**Fig. 5:**
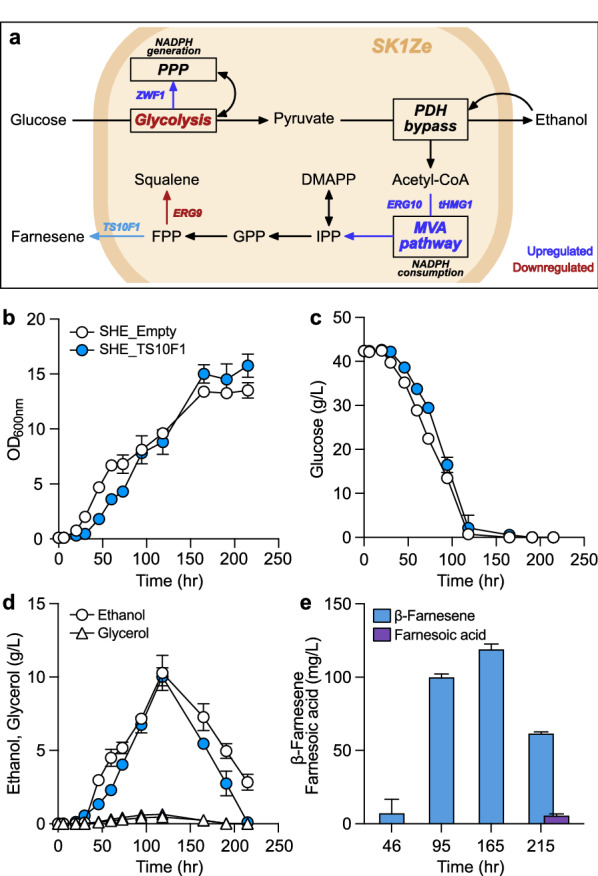
Microbial production of β-farnesene from glucose via TS10F1. **a** Overview of biosynthesis of β-farnesene from glucose by SHE_TS10F1. Upper glycolysis was downregulated, and *ZWF1* was overexpressed to shunt metabolic fluxes from glucose toward the pentose phosphate pathway to regenerate NADPH efficiently. *ERG9* was also downregulated to maximize FPP availability for β-farnesene biosynthesis. *ERG10* and *tHMG1* were episomally overexpressed with TS10F1 to enhance the flux through the MVA pathway. **b** − **d** Comparison of culture profiles of SHE_TS10F1 and negative control SHE_Empty on glucose, including cell growth (**b**), glucose consumption (**c**), and production of major extracellular metabolites (**d**). **e** β-Farnesene and farnesoic acid production through the glucose culture of SHE_TS10F1. SHE_Empty did not produce both compounds at detectable levels

We selected *S. cerevisiae* as a new host to test the potential of TS10F1 in microbial β-farnesene production on a sugar-based carbon source without noticeable innate β-farnesene biosynthesis. The TS10F1 ORF was codon-optimized and introduced into an engineered *S. cerevisiae* SK1Ze that limits downstream metabolism of the MVA pathway to accumulate FPP and shunting upper glycolysis toward the pentose phosphate pathway to efficiently regenerate NADPH, the key cofactor of the MVA pathway (Fig. 4a) [33]. TS10F1 was episomally overexpressed under a strong constitutive promoter (pRS426_P_CCW12_–TS10F1) together with acetyl-CoA C-acetyl transferase (*ERG10*) and truncated 3-hydroxy-3-methylglutaryl-co-enzyme-A reductase (*tHMG1*), the key enzymes of the MVA pathway (Fig. 5a). The resulting strain SHE_TS10F1 and negative control SHE_Empty carrying an empty pRS426_P_CCW12_ plasmid (Additional file 1: Table S1) exhibited similar growth patterns on glucose (Fig. 5b − d). SHE_TS10F1 produced β-farnesene efficiently in the glucose consumption phase (95 h, 3.89 ± 0.19 mg/g glucose), while SHE_Empty did not produce β-farnesene at detectable levels (Fig. 5e). SHE_TS10F1 exhibited relatively inefficient β-farnesene production during ethanol utilization, and the level of produced β-farnesene decreased until the ethanol depletion timepoint, 215 h (Fig. 5e). The detected highest β-farnesene titer was 119.74 ± 2.85 mg/L at 165 h. Interestingly, we discovered farnesoic acid from the 215 h sample analysis (Fig. 5e), implicating that innate promiscuous metabolic activities of *S. cerevisiae*, which were repressed during the glucose phase [34] or ethanol-dependently activated [35], converted β-farnesene into other metabolites including farnesoic acid in the SHE_TS10F1 culture.

### Future perspectives and challenges

This study demonstrated the potential of precursor toxicity-based functional metagenomic screening as a platform for bioprospecting novel TSs from uncultivated microbiota. We envision that this approach can be exploited further to identify additional novel TSs from other bioresources than the human fecal and soil samples used in this study. Furthermore, the selection pressure of functional metagenomic screening can be redesigned to discover other valuable metabolic functions residing in the metagenomes of uncultivated organisms, such as bioconversion of toxic compounds and decomposition of recalcitrant wastes [36, 37].

To enhance the functional metagenomic screening efficiency for TSs, improved control of false positives during the screening step would be required. Although the mutation rate of the LowMut strain is close to zero [24, 25], it could not eliminate spontaneous mutations resulting in toxicity mitigation without a TS activity (Fig. 2e). Additional safeguards, such as overexpression of *dnaE* [38], may reinforce the regulation of spontaneous mutagenesis and reduce the occurrence of false positives. Another area warranting further optimization is the low cloning efficiency of screened TS candidate ORFs which limited the number of characterizable candidates. We found indels destroying the expression cassette of TS candidates from few yielded constructs of the pET28b( +) cloning of TS candidates. These aberrant constructs with indels probably arose due to the metabolic consequence of the TS candidates [39]; they might be efficient enough to severely inhibit the *E. coli* growth even at the basal leaky expression level of the T7 system by depleting the native prenyl pyrophosphate pool without the overproduction of prenyl pyrophosphates via the MBIS pathway [40, 41]. Further studies with hosts and culture conditions enabling fine modulation of the prenyl pyrophosphate level, or the use of cell-free transcription–translation systems [42, 43], may be required to understand the potential toxicity of the screened candidates and to improve their cloning efficiency.

## Conclusions

The current study exploits functional metagenomics for screening TSs from metagenomic libraries based on the toxicity of prenyl pyrophosphates, the universal precursors of terpenes. The selection pressure of the functional metagenomic screening was optimized by controlling prenyl pyrophosphates biosynthesis via the MBIS pathway and mevalonate supplementation. An ORF, TS10F1, encoding a novel β-farnesene synthase from a human fecal metagenomic library was discovered through functional metagenomic screening and subsequent functional validation. Recombinant *S. cerevisiae* expressing the TS10F1 successfully produced β-farnesene from glucose. Our results highlight the potential of functional metagenomics as a unique approach for screening targeted metabolic pathways from uncultivated organisms from diverse environmental habitats.

## Methods

### Routine culture and cloning conditions

*E. coli* MegaX DH10B (Thermo Fisher Scientific, Waltham, MA) was used for routine cloning and selection pressure optimization. *E. coli* LowMut (Scarab Genomics, Madison, WI) was used as a host strain for the functional metagenomic screening. *E. coli* BL21(DE3) (Thermo Fisher Scientific) was used for in vivo enzyme tests and protein production. *S. cerevisiae* SK1Ze [33] was used for the terpene production from glucose. Details of strains used in this study are described in Additional file 1: Table S1.

*E. coli* strains were cultured in Luria Bertani medium (LB, 5 g/L yeast extract, 10 g/L tryptone, and 5 g/L NaCl). 34 μg/mL of chloramphenicol, 50 μg/mL of kanamycin, or 100 μg/mL of ampicillin were supplemented if required. Unless otherwise mentioned, *E. coli* strains were grown at 37 °C and 250 rpm. Yeast peptone medium with glucose (YPD, 10 g/L yeast extract, 20 g/L peptone, 20 g/L of glucose) was used for routine yeast cultures. Yeast transformants carrying auxotrophic plasmids were grown in a synthetic complete medium containing yeast nitrogen base, complete supplement mixture without histidine, leucine, and uracil (MP Biomedicals, Santa Ana, CA), and 40 g/L of glucose (SCD-3). The SCD-3 medium was adjusted at pH 5.5 using 50 mM potassium hydrogen phthalate buffer. Yeast strains were grown at 30 °C and 250 rpm unless otherwise mentioned.

Sanger sequencing was performed by Genewiz (Azenta Life Sciences, Chelmsford, MA), PCR was performed using Q5 HighFidelity DNA polymerase (New England Biolabs, Ipswich, MA), and codon-optimization and DNA fragment synthesis were accomplished through Integrated DNA Technologies (Coralville, IA). Plasmids and primers used in this study are described in Additional file 1: Tables S1 and S2.

### Optimization of the precursor toxicity

The MBIS operon [9] was expressed under the control of an IPTG-inducible promoter *P*_*lacUV5*_ in the pA5c backbone (pA5c–MBIS) to switch on growth inhibition by generating excessive prenyl pyrophosphates from mevalonate. The MBIS operon and pA5c backbone DNA fragments were prepared from pBbB5k–MBIS and pA5c–RFP by BamHI and EcoRI digestion. 1 M mevalonate solution was prepared by mixing 2 M mevalonate and 2 M KOH in a 1:1.02 ratio (v/v) and incubating at 37 °C for 30 min. Optimal mevalonate concentration for the screening process was determined through *E. coli* cultures with 200 μL of LB medium with kanamycin, chloramphenicol (LBKC), 0.5 mM of IPTG, and mevalonate with varied concentrations in clear bottom 96-well plates. *E. coli* growth was tracked every 5 min for 18 h at 37 °C using a Synergy H1 microplate reader (BioTek, Winooski, VT). The functional metagenomic screening was performed on solid media screening plates, composed of LBKC agar supplemented with 1 mM IPTG and 8 mM mevalonate, with plating of ~ 10^6^ colony forming units of functional metagenomic library cells or control strains.

### Simulation of precursor toxicity-based screening

Colony forming units (CFUs) of cell solutions of two positive controls (LowMut pA5c–MBIS pZE21–MsLim and LowMut pA5c–MBIS pZE21–AgBis) and one negative control (LowMut pA5c–MBIS pZE21–GFP) were measured via spotting assay on LBKC plates and frozen at − 80 °C with 20% glycerol. Two positive control strain cells were mixed with approximately 10^5^-fold more cells of negative control strain cells. The mixed cells were washed in sterilized PBS twice before spreading on screening medium plates, namely, LBKC with 1 mM IPTG and 8 mM mevalonate. To measure the actual CFUs of each strain in the mixture, each strain was processed in an identical way but with sterilized PBS instead of cell solutions of other strains and spread on LBKC without IPTG and mevalonate. Colonies on screening medium plates were identified via colony PCR amplifying the insert region of pZE21 (Additional file 1: Table S2 and Fig. S2a, d). The recovery rate of each strain was calculated using the CFUs on screening medium plates and CFUs on LBKC.

### Metagenomic library

Metagenomic library plasmids used in this study were previously prepared by cloning sheared metagenomic DNA fragments (2–5 kb) of human fecal specimens collected from El Salvador (40101, 40203, and 40301) or soil samples from Michigan (S18) [44, 45]. The backbone plasmid pZE21 constitutively expresses ORFs under the control of *P*_*LtetO-1*_ in both DH10B and LowMut host strains, both of which lack the tetracycline repressor [46].

### Characterization of TS candidates

Metagenomic insert region sequences of the selected library plasmids were identified via Sanger sequencing. The sequencing was performed initially with primers 5253 and 5254 and continued with new primers designed based on the initial sequencing results. Amino acid sequences of the ORFs were analyzed using protein BLAST (https://blast.ncbi.nlm.nih.gov), ConSurf [47], and PyMOL 2.5.2 [48]. Selected ORFs from the library plasmids were PCR amplified and introduced into pET28b( +) (MilliporeSigma, St. Louis, MO) with N-terminal or C-terminal hexa histidine-tag for in vivo functionality tests and protein production.

To investigate putative products of the screened TS in vivo, BL21(DE3) transformed with pA5c–MBIS and the TS expressing plasmid was recovered in 5 mL LBKC at 37 °C overnight. Recovered cells were harvested, washed in sterilized PBS, and inoculated into 5 mL EZ rich defined medium (Teknova, Hollister, CA) with kanamycin and chloramphenicol in a glass tube at initial OD600nm 0.1. 12 mM mevalonate and 0.1 mM IPTG were supplemented at OD600nm 0.5. After adding 0.5 mL filtered dodecane, the cultures were continued at 20 °C. Dodecane layer was collected at 10-, 24-, 34-, and 48-h timepoints.

To perform in vitro enzyme assays, target TS protein was overproduced in BL21(DE3) without pA5c–MBIS. The production culture was performed in 200 mL LB with kanamycin (LBK) in a 1 L baffled flask at initial OD600nm 0.1, and IPTG was added to a final concentration of 0.1 mM at OD600nm 0.5. Then the culture was continued at 20 °C for 18 h. Target TS protein was extracted and purified using HisTALON™ Gravity Columns Purification Kit (Takara Bio, San Jose, CA). Purified protein samples were desalted using Amicon® Ultra-0.5 10 K (MilliporeSigma) and concentrated to 1 μg/μL in 2X reaction buffer (80 mM MgCl2, 8 mM MnCl2, 8 mM DTT, 50 mM HEPES, pH 7.2). The enzyme reaction was initiated in a glass vial by combining 100 μL of the concentrated enzyme solution with the mixture of 100 μL of the mixture of a potential substrate (GPP or FPP, 0.1 mM) and a cofactor (NADH, NADPH, 0.1 mM, or water) in water. To rule out the spontaneous conversion of substrates and define the baseline chromatogram for the gas chromatography–mass spectrometry (GC–MS) analysis of reaction products (see “Analysis of microbial metabolites and enzyme reaction products” section), we also prepared negative control reaction mixtures by adding 2X reaction buffer instead of the enzyme solution. After brief vortexing, 200 µL filtered dodecane was added to capture enzyme reaction products, and the dodecane layer was sampled at 0  and 3 h timepoints.

### Terpene production in the engineered yeast

A selected TS ORF was codon-optimized, amplified, and introduced into pRS426_P_CCW12_ [33] for constitutive expression in yeast. *tHMG1* and *ERG10* coding sequences were amplified from *S. cerevisiae* S288C genomic DNA and introduced into pRS423_P_TDH3_ and pRS425_P_TEF1_ (EUROSCARF), respectively. The lithium acetate/single strand carrier DNA/polyethylene glycol method [49] was used for yeast transformations.

Yeast strains were recovered from frozen glycerol stocks on SCD-3 plates in a 30 °C incubator. A single colony of each strain was inoculated in 5 mL of SCD-3 broth and grown at 30 °C and 250 rpm overnight as a seed culture. For precultures, seed cultures were transferred into fresh 5 mL liquid SCD-3 at initial OD600nm 0.5 and incubated for additional 6 h. Main cultures were performed with 50 mL of SCD-3 in a 250 mL baffled flask at initial OD600nm 0.1. After inoculation, 5 mL of filtered dodecane was added to capture β-farnesene, and flasks were incubated at 30 °C, 300 rpm. Culture broth (bottom) was collected for tracking yeast cell growth, glucose, and soluble extracellular metabolites. The dodecane layer (top) was analyzed to quantify the targeted terpene and its hydrophobic derivatives. Intracellular hydrophobic metabolites were extracted from the cell pellets following the procedure previously described [50].

### Analysis of microbial metabolites and enzyme reaction products

Terpenes and terpene derivatives collected in dodecane were analyzed through GC–MS composed of Agilent 7820A GC equipped with an HP-5 ms column and 5977E MSD (Agilent Technology, Wilmington, DE). The split ratio was 1:1, and filaments were heated at 250˚C for 2 min. The initial temperature of the column oven was 100˚C for 3 min, followed by a 10˚C/min ramp to 280˚C for 3 min. Dodecane samples from in vitro enzyme assays and yeast cultures were analyzed using 7890 GC and 5977B MSD (Agilent Technology) in identical conditions. Terpenes were identified using the National Institute of Standards and Technology (NIST) database. The identification results were confirmed and quantified using corresponding standard chemicals.

Microbial cell density in the culture broth was monitored by absorbance at 600 nm (A600) using DiluPhotometer (Implen, Westlake Village, CA). Glucose and extracellular metabolites in culture broth were quantified by HP 1050 HPLC system equipped with 1100 Refractive Index Detector (Agilent Technology), Rezex ROA Organic Acid H + (8%) column (Phenomenex, Torrance, CA), and 0.005 N H2SO4 as a mobile phase (0.6 mL/min and 50 °C).

## Supplementary Information

**Additional file 1.** Additional data.

## Data Availability

All assembled sequences have been deposited to Genbank and are available via the BioProject PRJNA835076 (https://www.ncbi.nlm.nih.gov/bioproject/835076).

## Abbreviations

MEP pathway: 2-C-methyl-D-erythritol 4-phosphate pathway
MVA pathway: Mevalonate pathway
TS: Terpene synthase
AgBis: *Abies grandis* Bisabolene synthase
GFP: Green fluorescent protein
MsLim: *Mentha spicata* Limonene synthase
IPTG: Isopropylthio-β-galactoside
ORF: Open reading frame
MATE: Multidrug and toxic compound extrusion
AEC: Auxin efflux carrier
ABC: ATP-binding cassette
FPP: Farnesyl pyrophosphate
GPP: Geranyl pyrophosphate
