# Supplementary material for: Functional mining of novel terpene synthases from metagenomes
Source: Biotechnol Biofuels Bioprod. 2022 Oct 8;15:104. doi: 10.1186/s13068-022-02189-9 (PMC9548185; doi:10.1186/s13068-022-02189-9)
Supplement: Supplementary file 1 — Additional file 1. Additional data. [file 13068_2022_2189_MOESM1_ESM.pdf]

Supplementary data for

Functional mining of novel terpene synthases from metagenomes

Suryang Kwak[1,2]*, Nathan Crook[1,2,3]*, Aki Yoneda[1], Naomi Ahn[1], Jie Ning[1], Jiye Cheng[1,2], Gautam Dantas[1,2,4,5]**

[1] Edison Family Center for Genome Sciences & Systems Biology, Washington University School of Medicine in St. Louis, MO, USA 63110

[2] Department of Pathology and Immunology, Washington University School of Medicine in St. Louis, MO, USA 63110

[3] Department of Chemical and Biomolecular Engineering, North Carolina State University, Raleigh, NC, USA 27695

[4] Department of Biomedical Engineering, Washington University in St. Louis, St. Louis, MO, USA 63110

[5] Department of Molecular Microbiology, Washington University School of Medicine in St. Louis, MO, USA 63110

* **Contributed equally** to this work

** **Corresponding author**: Gautam Dantas (dantas@wustl.edu)

This PDF file includes:

- 2 Supplementary Tables
- 5 Supplementary Figures
- Supplementary Texts: sequence information of 10 terpene synthase candidates

SUPPLEMENTARY TABLES

Table S1. Strains and plasmids used in this study.

Name	Description	Reference
Strains		
DH10B	F⁻ *mcrA* Δ(*mrr-hsdRMS-mcrBC*) Φ80*lacZ*Δ*M15* Δ*lacX74 recA1 endA1 araD139* Δ(*ara leu*)*7697 galU galK* λ⁻ *rpsL nupG*	Thermo Fisher Scientific
LowMut	https://www.scarabgenomics.com/product/clean-genome-lowmut	Scarab Genomics
BL21(DE3)	F⁻ *ompT gal dcm lon hsdS$_B$*($r_B^-m_B^-$) λ(DE3 [*lacI lacUV5-T7p07 ind1 sam7 nin5*]) [*malB*$^+$]$_{K-12}$(λ^S)	Thermo Fisher Scientific
BM_TS10F1	BL21(DE3) pA5c-MBIS pET28b-TS10F1	This study
BM_AgBis	BL21(DE3) pA5c-MBIS pET28b-AgBis	This study
BM_Empty	BL21(DE3) pA5c-MBIS pET28b	This study
SK1Ze	*S. cerevisiae* D452-2, substitution of P_{SYH1} for P_{ERG9} and P_{TDH3} for P_{ZWF1}	(Kwak et al., 2019)
SHE_TS10F1	SK1Ze pRS423_P_{TDH3}-tHMG1 pRS425_P_{TEF1}-ERG10 pRS426_P_{CCW12}-TS10F1	This study
SHE_Empty	SK1Ze pRS423_P_{TDH3}-tHMG1 pRS425_P_{TEF1}-ERG10 pRS426_P_{CCW12}	This study
Plasmids		
pA5c-RFP	Source of pA5c backbone (p15A origin, *Cm^R*)	FZ105*
pBbB5k-MBIS	Source of P_{lacUV}-MBIS (*Kan^R*)	FZ239*
pW1a-AgBis	Source of AgBis (*Amp^R*)	FZ260*
pA5c-MBIS	p15A origin, *Cm^R*, P_{lacUV}-MBIS	This study
pZE21	colE1 origin, *Kan^R*, $P_{LtetO-1}$	(Lutz and Bujard, 1997)
pZE21-AgBis	colE1 origin, *Kan^R*, $P_{LtetO-1}$-AgBis	This study
pZE21-MsLim	colE1 origin, *Kan^R*, $P_{LtetO-1}$-MsLim	This study

pZE21-GFP	colE1 origin, Kan^R, $P_{LtetO-1}$-GFP	This study
pET28b(+)	pBR322 origin, Kan^R, P_{T7}	MilliporeSigma
pET28b-AgBis	pBR322 origin, Kan^R, P_{T7}-AgBis	This study
pET28b-TS10F1	pBR322 origin, Kan^R, P_{T7}-TS10F1	This study
pRS423_P_{TDH3}	2μ origin, *HIS3*, P_{TDH3} and T_{CYC1}	EUROSCARF
pRS425_P_{TEF1}	2μ origin, *LEU2*, P_{TEF1} and T_{CYC1}	EUROSCARF
pRS426_P_{CCW12}	2μ origin, *URA3*, P_{CCW12} and T_{CYC1}	(Kwak et al., 2019)
pRS423_P_{TDH3}-tHMG1	pRS423_P_{TDH3} expressing *tHMG1***	This study
pRS425_P_{TEF1}-ERG10	pRS425_P_{TEF1} expressing *ERG10***	This study
pRS426_P_{CCW12}-TS10F1	pRS426_P_{CCW12} expressing TS10F1 ORF***	This study

* Gifts from Dr. Fuzhong Zhang, Department of Energy, Environmental & Chemical Engineering, Washington University in St. Louis
** Cloned from *S. cerevisiae* S288C
*** Codon optimized for *S. cerevisiae*

Table S2. Primers used in this study (additional sequencing primers for the library sequencing were omitted).

Description	Direction	Sequence
AgBis pZE21 cloning (blunt end)	Forward	ATGGCGGGTGTTTCTGC
	Reverse	TTACAGCGGCAGCGGTTC
GFP pZE21 cloning (blunt end)	Forward	ATGAGCAAAGGAGAAGAACTTTTCACTG
	Reverse	TTATTTGTAGAGCTCATCCATGCCATG
MsLim pZE21 cloning	Forward	aaaggtaccATGCGTCGCAGTGGTAATTA
	Reverse	tttGGATCCTTAGGCGAAAGG
pZE21 initial sequencing	Forward	CACATCAGCAGGACGCACTGACC
	Reverse	AGGAGAGCGTTCACCGACAAACAACAG
pZE21 colony PCR confirmation	Forward	GCGTATCACGAGGCCCTTTC
	Reverse	GGCGGCGGATTTGTCCTACT
AgBis pET28b(+) cloning	Forward	acacatATGGCGGGTGTTTCTGC
	Reverse	acaggatccTTACAGCGGCAGCGGTTC
TS10F1 pET28b(+) cloning	Forward	acacatATGAAAAAGACAACTCTACTTCTTG
	Reverse	acaggatccTTATAGATCCAGCGTCCTTACCTC
tHMG1 cloning for yeast expression	Forward	taaactagtaaaaca*atgg*ctGCAGACCAATTGGTGAAAACT
	Reverse	acagtcgacTTAGGATTTAATGCAGGTGACG
ERG10 cloning for yeast expression	Forward	taaactagtaaaacaATGTCTCAGAACGTTTACATTGTATC
	Reverse	acagtcgacTCATATCTTTTCAATGACAATAGAGGAAG
TS10F1 cloning for yeast expression	Forward	taaactagtaaaacaATGAAAAAGACAACTCTACTTCTTGCACG
	Reverse	acactcgagTTATAGATCCAGCGTCCTTACCTCTG

Restriction sites were underlined. Binding sequences were uppercased. Artificial start codon for *HMG1* truncation was italicized.

SUPPLEMENTARY FIGURES

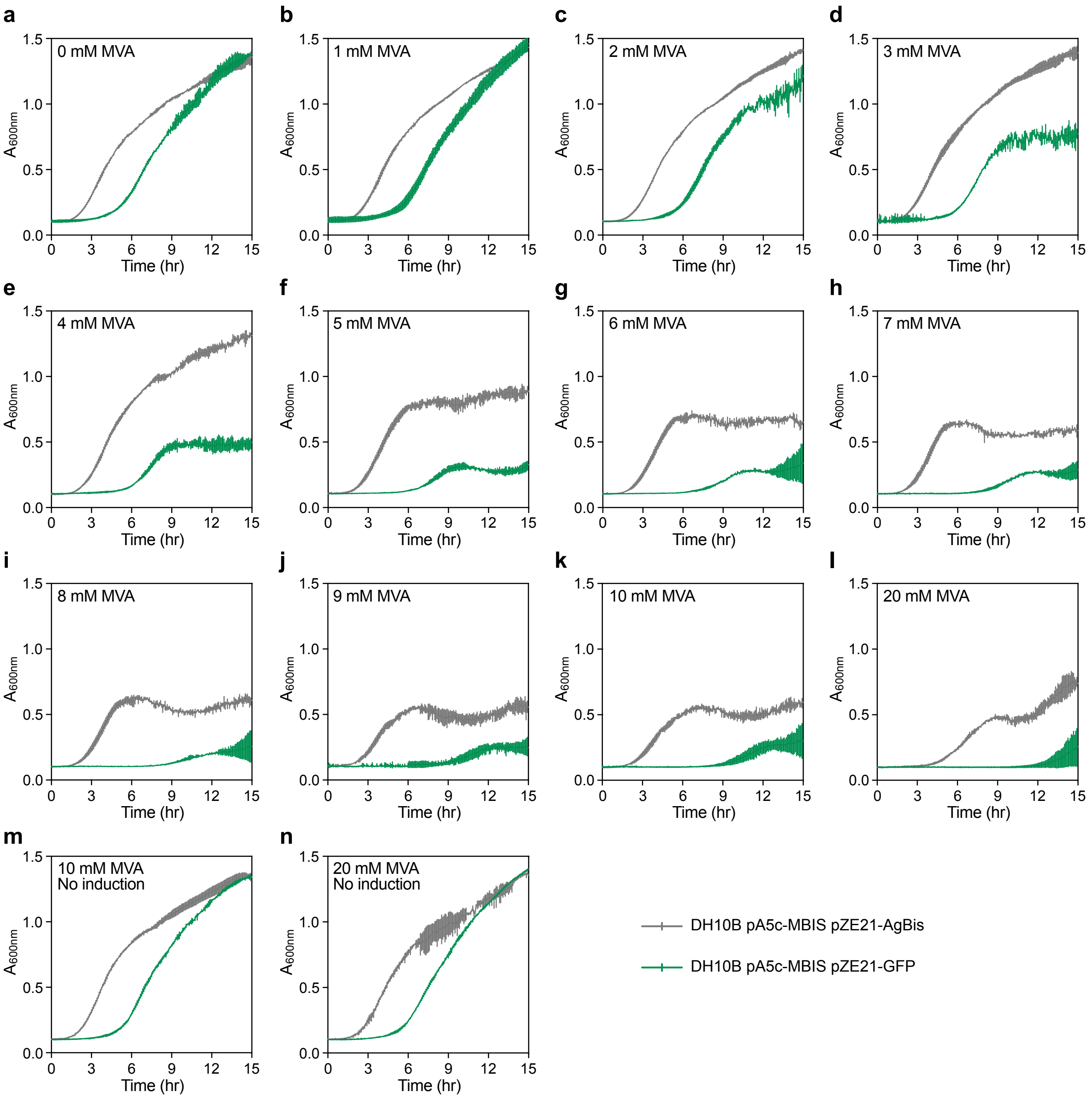

Figure S1. Growth profiles of the positive (gray) and negative (green) control strains on varied mevalonate (MVA) concentrations. (a−l) Culture medium included 0.5 mM IPTG to induce the MBIS operon. (m and n) Cultures without IPTG induction. Regardless of MVA concentration in the medium, both strains exhibited similar growth patterns to those of the cultures without MVA supplementation (a).

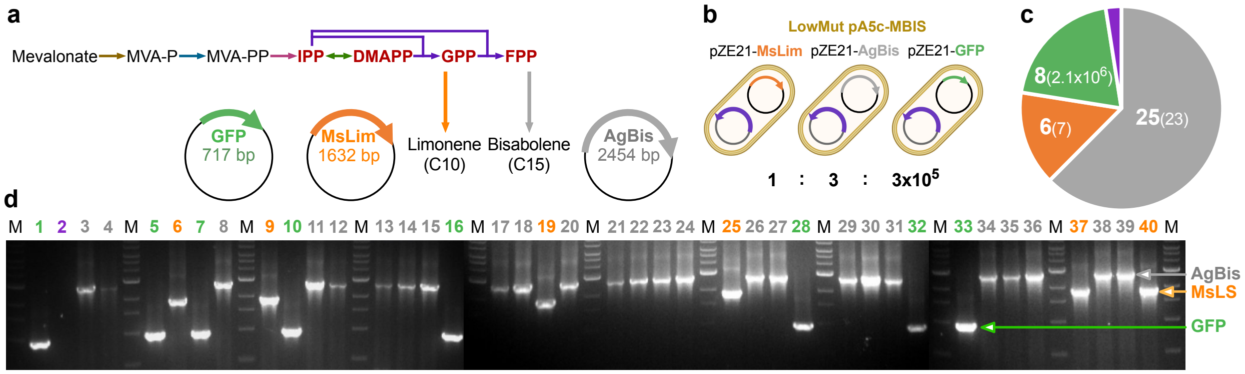

Figure S2. The simulation of precursor toxicity-based screening. (a−b) An artificial library mixture was prepared with two positive control pZE21 plasmids expressing model monoterpene synthase (MsLim) and sesquiterpene synthase (AgBis) and one negative control pZE21 plasmid expressing GFP. (a) MsLim and AgBis are well-characterized terpene synthases consuming GPP and FPP, respectively, as substrates. The plasmids were transformed into LowMut pA5c-MBIS strain, and the resulting constructs were mixed as described in (b). (c−d) Total 40 colonies were screened on the screening medium plate and identified via colony PCR of the insert region of pZE21 (Table S2). (c) Numbers in bold represent the total CFUs of each strain on screening medium plates of the artificial library mixture, and small numbers in parentheses were CFUs of each strain measured on normal LBKC plates after identically processing each strain with sterilized PBS instead of cell solutions of other two strains. (d) Gel electrophoresis of the colony PCR products. PCR amplification failed from one colony (no. 2, highlighted in purple).

Library	Contig	ORF (bp)	BLAST
**40101**	TS01	TS01F1 (609)	MATE transporter
	TS05	TS05F1 (780)	Unknown
	TS06	TS06F1 (1620)	Putative transposase
	TS11	TS11F1 (1422)	Unknown
		TS11F2 (984)	MATE transporter
	TS20	TS20F1 (1620)	= TS06F1
	TS24	TS24F1 (630)	Unknown
	TS44	Too short	
	TS46	No ORF	
	TS48	Too short	
**40203**	TS30	TS30F1 (1336)	Unknown
		TS30F2 (1149)	Transcriptional regulator
	TS32	TS32R1 (492)	ABC transporter
	TS33	Too short	
	TS34	TS34F1 (1336)	Transcriptional repressor
	TS36	TS36R1 (540)	Histidine phosphatase family protein
	TS37	TS37F1 (639)	MATE transporter
	TS40	TS40F1 (615)	MATE transporter
		TS40R1 (540)	= TS36R1
	TS41	No priming	
	TS43	TS43R1 (540)	= TS36R1
	TS47	No priming	
**40301**	TS02	TS02F1 (756)	AEC transporter
	TS04	TS04F1 (786)	Phosphatase family protein
		TS04F2 (1573)	Putative DNA glycosylase
	TS09	TS09F1 (705)	Phosphoglycerate mutase family protein
	TS10	TS10F1 (513)	Histidine phosphatase family protein
	TS12	TS12F1 (513)	= TS10F1
	TS17	TS17F1 (513)	= TS10F1
	TS18	TS18F1 (618)	Histidine phosphatase family protein
**S18**	TS35	No insert	

Figure S3. The outcome of the first precursor toxicity-based functional metagenomic screening. Blue, novel terpene synthase candidates (see Supplementary Texts for detailed information); green, duplicate ORFs; red, false positives.

```
        TS10F1  MKKTTLLLARHGETVDNANRIMQGQTQGRLNENGIRQAEELAGRMKDRKIDAFVASDLKRSVDTCRIVAA  70
                   T L L RHGET DNAN+IMQGQT GRLN +GI QA+E+A ++ ++  DAFV+SDLKRS+DTCRI+A
WP_044075392.1  M--TRLYLTRHGETYDNANKIMQGQTPGRLNNHGIEQAKEVAKKLAEKHFDAFVSSDLKRSIDTCRIIAE  68

        TS10F1  PHHAEVHTTQLLRERDWGGFTGRYIPDLKGETWPDDVESLDALKLRAQRFLDYIKREYSGQTVFAVGHGI  140
                PHHA+V TT LLRERDWG FTGRYIPDLK E WPDDVESLD LK RA+ FL++IKREY GQTV AVGHGI
WP_044075392.1  PHHADVRTTPLLRERDWGSFTGRYIPDLKDEVWPDDVESLDTLKARARDFLEFIKREYPGQTVLAVGHGI  138

        TS10F1  TNKAIQAVYYNKEMKDIEKMANAEVRTLDL  170
                 NKAIQAVYYNKEMKD++KM NAEVR LDL
WP_044075392.1  INKAIQAVYYNKEMKDVQKMTNAEVRVLDL  168
```

Figure S4. Comparison of amino acid sequences of TS10F1 and a histidine phosphatase family protein of *Prevotella pectinovora* (WP_044075392.1). Green-highlighted amino acid residues represent catalytic core regions. Sky blue, correct match; blue, functional equivalence; plum, not similar amino acid.

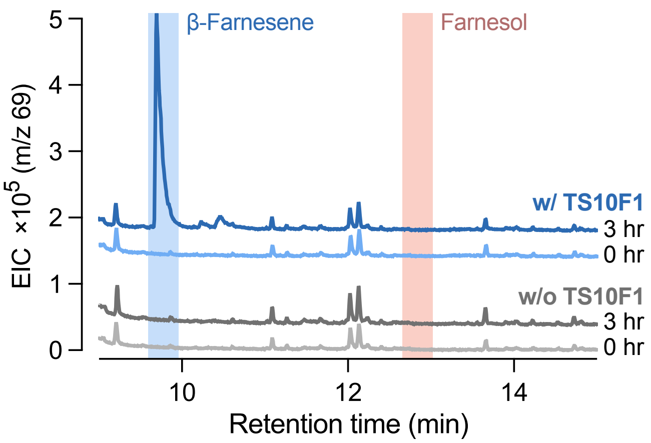

Figure S5. Magnified chromatograms of *in vitro* enzyme assay samples from the actual reaction initiated by adding purified and desalted TS10F1 (top, blue) and negative control reaction without TS10F1 (bottom, gray).

SUPPLEMENTARY TEXTS

Sequence information of terpene synthase candidates

1. TS04F1 (Library 40301)
>DNA sequence
ATGGGCACGACGATGCTCCCCGGCGTGGGCTGCTCCGCGATTGGCGTGTCGGAAGTGGAAAACGACCGTT
GCCTTACCGACCTGTGTGCCCAGCCTGAGCTCAGCAAGCGCGGATACAAGTTCTGCCATGTCGAGGGTCC
CGACCGCCGTGGCATCGACTGCGCCCTTATCTACAATCCGTCGCTCTTTTCCGTCAAGGCTTATAGGCTC
TACCCTTATGTGCCCACCGAGAAGCAAGACTCGCTCTTCCGCACCCGTGGCTTCTTTGCCGTGAGCGGTG
AGATGGCTGGCGAACACGTGGTGATAATCGTCAACCACCTGCCGAGCCGCTTCAATGTCAGCTACTTCCG
CGAGGTGGGCGCCGCGCAGGTGAAGGCGTTGAAAGACAGGATCCTCGGCCTTGACCCTAAGGCGAAAGTC
ATAGTGATGGGCGACATGAACGACGACCCTACCAACAAGAGCATGCATGAGGTGCTCTCTGCTAAGGAGG
AGGCGGACATGGTTGGCAAGGACGACATGTATAACCCGTGGTACAACGTGCTTGCCAAGCAAGGCACCGG
CACGCTGCAATATCAAGGCTCATGGAACCTTTTCGACCAGATAATATTGTCGCCAAGCCTTATAAACAAG
AACGGGGAAAAGGATTTCACGACGCTTAAATACTTCAAGTACGAGGTTCAGCGCATGCCTTACCTCTTCC
AGACGGAAGGCAAGTACAAGGGAGGCACCAAGCGCACCACGGCCGGAGGCGTTTGGCTCGACGGCTTCTC
TGACCACCTGCCCACCGTGGTGTATCTCGTGAAAGAACAGCGCGAGAAGAAAGACAAGGCGTTGAAGGAA
AGCGTGTTCGCCCTGCCCGAGTACACGGAGGCTGAGCTGAGGAACATCGCCTCGTCGGAGGCTGAGCAGA
AGGCAAGGGTGGAGAAAGACAAGAATAAGAAAGACTGA
>Amino acid sequence
MGTTMLPGVGCSAIGVSEVENDRCLTDLCAQPELSKRGYKFCHVEGPDRRGIDCALIYNPSLFSVKAYRL
YPYVPTEKQDSLFRTRGFFAVSGEMAGEHVVIIVNHLPSRFNVSYFREVGAAQVKALKDRILGLDPKAKV
IVMGDMNDDPTNKSMHEVLSAKEEADMVGKDDMYNPWYNVLAKQGTGTLQYQGSWNLFDQIILSPSLINK
NGEKDFTTLKYFKYEVQRMPYLFQTEGKYKGGTKRTTAGGVWLDGFSDHLPTVVYLVKEQREKKDKALKE
SVFALPEYTEAELRNIASSEAEQKARVEKDKNKKD-
>BLAST result
Endonuclease [Prevotella sp.] HCC88143.1 (Identity: 90%)
Endonuclease/exonuclease/phosphatase family protein [Prevotella multisaccharivorax] WP_007575158.1 (identity: 80%)

2. TS04F2 (Library 40301)
>DNA sequence
ATGCCGACGCTTGACATAGAAAGCCATCCCTTTGAGCCTTTCTTGCCCAAGACGGCCCGGTTGCTGATGC
TCGGTACCTTCCCGCCATCGCCAAAACGCTGGTGCATAGACTGGTATTATCCCAACTACACTAACGACAT
GTGGCGCATCATGGGGCTGTGCTTCTTTGGCGACAAGCTTCATTTCGTGGACGAGGGCAACAAGACCTAT
CGCCTCGACGACTTGAAGGCGTTTCTCTCGGAGAGGGGCATCGCCATTTTCGACACTTGCTTGCGAATCA
GGCGTACCAAGGGGACGGCATCAGACAAGGACTTGGAAGTGGTGGAGAGGGCAGACCTCGACGCCATGCT
CACGGCCTTGCCGCTATGTCGTGGTGTGCTCACGGCAGGCCGGCTGGCCACCAAAATCTTCATGGAGCAT
TATGGCATAGGCCAGAAGGCCAACGACTTGAAGATGGGCGGGCATTTGGATTTCGACTTCCATGGCAGGG
CGATAAGCCTGTACAGGCAGCCAAGCTCAAGCCGTGCCTATCCCATGAAGCTTGAGGACAAGGCCGCGTA
TTATGACAACATGTTCAGGGAATTGGGCATTTTATAA
>Amino acid sequence

MPTLDIESHPFEPFLPKTARLLMLGTFPPSPKRWCIDWYYPNYTNDMWRIMGLCFFGDKLHFVDEGNKTY
RLDDLKAFLSERGIAIFDTCLRIRRTKGTASDKDLEVVERADLDAMLTALPLCRGVLTAGRLATKIFMEH
YGIGQKANDLKMGGHLDFDFHGRAISLYRQPSSSRAYPMKLEDKAAYYDNMFRELGIL-
>BLAST result
DNA glycosylase [Prevotella sp.] HCC88144.1 (Identity: 87%)

3. TS05F1 (Library 40101)
>DNA sequence
ATGAGGCGGCAGAGTTCGCGCGCCACCTGCTCGAGAAGTCCTTCGAGGCGCCCACGCCCTCGTACGTGCG
GGAGCCGGGCTTTCAAGATCTCATGGGCAACCGGGCTGCCGCAGAGCCGCAGACGCGCTTTCTCGCATGC
GGGGATTCCGATTTGGAAGAGCCTGCAGATGCGAGCGGCGACGGGGCGGCGGAGGATCCGCTCGCCAAGC
CGCGCTGCAACGAGGAATGGTACTCTTTCATCTGTGCCGAGCTCTCGCTCGACAGCCTGCCCGACTACCA
CTACATCTACGAGCCGGAAGACGGGACCTATCGCGTGCGGGCGCTCAACCACCGCCTCGACGGGCATCCT
GGGCTCGCGTACCTGGAAGCGCGCAAGAATGCCCAGGTCGCCTTTGCCGAGCAGTACGATTTCCTGCTCA
TGCAGCGCCTGTATTCGGGGACGTGCGAGCCCGTGTCTCGCTACGAGTGCGACATGCTCTCGGACCGCAT
CTGGTTCGGTTCTTTCTCGGATTCgAATGACGTCTCGGTGGAAAGCGACAAGACCGGGGAGTATCTCAAA
GAGGTGCGCGAGGCCTACGAAAACGCCGCGCGGCAAGATCCTACGTCCGTGCTGCCGCGCGCGGTGTTCG
ACCGTCTGTGCGAATGGTTCCGCCGCCGTTCGGAGGGCGACCTCGACGCGTGCGCGCGCGAGATCGACGA
TGCCTGCGCCAAGGTGCTCTCTCCTTTCAGGTATGAGTTTTCCGCCAGCTTCGACTACGAGCGCAAACTC
GCCCGGATAA
>Amino acid sequence
MRRQSSRATCSRSPSRRPRPRTCGSRAFKISWATGLPQSRRRAFSHAGIPIWKSLQMRAATGRRRIRSPS
RAATRNGTLSSVPSSRSTACPTTTTSTSRKTGPIACGRSTTASTGILGSRTWKRARMPRSPLPSSTISCS
CSACIRGRASPCLATSATCSRTASGSVLSRIRMTSRWKATRPGSISKRCARPTKTPRGKILRPCCRARCS
TVCANGSAAVRRATSTRARARSTMPAPRCSLLSGMSFPPASTTSANSPG-
>BLAST result
No significant similarity found

4. TS09F1 (Library 40301)
>DNA sequence
ATGTCGCCAATGGCTGCGCTAAGCAACAGATTGTCCATAATGAAAAGGTGTTTGTGGGGGATAGAATTGT
TTCTGTTTCGTAAGCAAAACTTACATTGGCAAAATTATCAAAAAATCGCGAATTATCATTACTTTTGCAG
TCGGAAAAAAAGAAAAGGAACAAAGAAAATAAAAAAAGAAAGAAAACAAGCAATGACACATACAACCCTT
TTGCTCACGCGCCACGGCGAAACGGTGGACAACGTGAACCAGATTATGCAAGGACAGACTCAAGGACAGC
TCACCACCCACGGACGCGAACAGGCTGAGGTGGTGGCGAAAGAGATGGCAGACAGGCAGATCGACGCTTT
CGTGTCGAGCGATCTCAAACGCGCCATCGACACTTGCCACATCATAGCCCTACCCCACAAAGCGCCTGTT
GTGACCACGCCTCTGCTGCGCGAGCGCGACTGGGGAGGTTTCACAGGGCGTTACATACCCGACCTTAAAG
GTGAGCCATGGCCTGACGACATTGAGACGATGGACGACCTGAAAGCGCGTGCGGCACGCTTCATCGAGTT
CATACGCCACAGCTATCCCGGAAAGACGGTCTTGGCCGTGGGCCACGGCATCATTAACAAGGCCATACAA
AGCGTCTATTATGACAAGCCCACAAACAAGATTGTGCCGATGAAAAACGCTGAGGTGCGCGTGCTGGAGC
TTTGA
>Amino acid sequence
MSPMAALSNRLSIMKRCLWGIELFLFRKQNLHWQNYQKIANYHYFCSRKKRKGTKKIKKERKQAMTHTTL
LLTRHGETVDNVNQIMQGQTQGQLTTHGREQAEVVAKEMADRQIDAFVSSDLKRAIDTCHIIALPHKAPV
VTTPLLRERDWGGFTGRYIPDLKGEPWPDDIETMDDLKARAARFIEFIRHSYPGKTVLAVGHGIINKAIQ
SVYYDKPTNKIVPMKNAEVRVLEL-

>BLAST result
Phosphoglycerate mutase family protein [Marseilla massiliensis]
WP_205105982.1 (Identity: 75%)

5. TS10F1 (Library 40301)
>DNA sequence
ATGAAAAAGACAACTCTACTTCTTGCACGCCACGGAGAAACCGTGGATAACGCCAACCGGATTATGCAGG
GACAGACACAAGGAAGGCTCAACGAAAACGGAATCAGACAGGCGGAAGAACTTGCAGGAAGAATGAAAGA
CAGAAAGATTGATGCTTTCGTGGCGAGCGACCTAAAACGCTCCGTAGACACTTGCCGCATCGTGGCTGCG
CCACACCATGCGGAAGTACATACCACACAACTGCTGAGAGAAAGAGACTGGGGAGGCTTCACCGGCAGAT
ACATTCCCGACCTGAAAGGAGAAACATGGCCAGACGACGTGGAATCCTTGGACGCCCTGAAGCTGCGCGC
ACAAAGATTTCTGGACTATATCAAAAGGGAATATTCCGGACAGACAGTCTTTGCCGTAGGCCACGGTATA
ACAAACAAAGCCATACAGGCCGTCTACTACAACAAAGAGATGAAAGACATCGAAAAGATGGCTAATGCAG
AGGTAAGGACGCTGGATCTATAA
>Amino acid sequence
MKKTTLLLARHGETVDNANRIMQGQTQGRLNENGIRQAEELAGRMKDRKIDAFVASDLKRSVDTCRIVAA
PHHAEVHTTQLLRERDWGGFTGRYIPDLKGETWPDDVESLDALKLRAQRFLDYIKREYSGQTVFAVGHGI
TNKAIQAVYYNKEMKDIEKMANAEVRTLDL-
>BLAST result
Histidine phosphatase family protein [Prevotella pectinovora]
WP_044075392.1 (Identity: 75%)

6. TS11F1 (Library 40101)
>DNA sequence
ATGCCCGCAGCGGCGTACTCCTTCGTTGGTCATATCGTAAAAATAGGAGTGCACCACCCGGGCAATGCCG
TTGTGCGCCACCAGCAGGTAGGTCTTGCCGGTGTCCGCCTTCAATTCGTCCAGCAGGTTGTAGATGCGCT
GCGCCAGCTGCATCATGCTTTCGCCGCCGTCATAGCGGTCGGCAAAGTGGGTCTTGGAGATGCGGAACTC
CGCACCGTCCCGGGGCGTGCCCTCGTATTTGCCAAAGCACTGCTCCCGCAGACGGGGCTCGCAGCGGGCA
GGCAGACCGGTAGCGGCGGCAATGGCTTTTGCGGTGTCGGCGGCGCGGGAAAGGGGAGAGTACAAGATCT
CGTCGATGTGCAGGCCGCTGTCCCGGACAAGTTCGCCCAGCTGCCGCGCCTGCTCCTGCCCGCGGGCGGT
CAGCGGGCTGTCGGTCATGCCGCAGATCTTATTTTCCACGTTCCATACCGTCTCGCCGTGACGGGTGAAA
TAGATATTATGCATAAAAATACTCCTTACAGCTTGTGCAGGGTGCCATCCGGCAGCAGACAAACGGTGTC
TCCTTCGTCGATGCCCAGACGCGTCAGTTCTTTGTCCGGGTAG
>Amino acid sequence
MPAAAYSFVGHIVKIGVHHPGNAVVRHQQVGLAGVRLQFVQQVVDALRQLHHAFAAVIAVGKVGLGDAEL
RTVPGRALVFAKALLPQTGLAAGRQTGSGGNGFCGVGGAGKGRVQDLVDVQAAVPDKFAQLPRLLLPAGG
QRAVGHAADLIFHVPYRLAVTGEIDIMHKNTPYSLCRVPSGSRQTVSPSSMPRRVSSLSG-
>BLAST result
No significant similarity found

7. TS18F1 (Library: 40301)
>DNA sequence
ATGTCGCCAATGGCTGCGCTAAGCAACAGATTGTCCATAATGAAAAGGTGTTTGTGGGGGATAGAATTGT
TTCTGTTTCGTAAGCAAAACTTACATTGGCAAAATTATCAAAAAATCGCGAATTATCATTACTTTTGCAG
TCGGAAAAAAAGAAAAGGAACAAAGAAAATAAAAAAAGAAAGAAAACAAGCAATGACACATACAACCCTT

TTGCTCACGCGCCACGGCGAAACGGTGGACAACGTGAACCAGATTATGCAAGGACAGACTCAAGGACAGC
TCACCACCCACGGACGCGAACAGGCTGAGGTGGTGGCGAAAGAGATGGCAGACAGGCAGATCGACGCTTT
CGTGTCGAGCGATCTCAAACGCGCCATCGACACTTGCCACATCATAGCCCTACCCCACAAAGCGCCTGTT
GTGACCACGCCTCTGCTGCGCGAGCGCGACTGGGGAGGTTTCACAGGGCGTTACATACCCGACCTTAAAG
GTGAGCCATGGCCTGACGACATTGAGACGATGGACGACCTGAAAGCGCGTGCGGCACGCTTCATCGAGTT
CATACGCCACAGCTATCCCGGAAAGACGGTCTTGGCCCGTGGGCCACGGCATCATTAA
>Amino acid sequence
MSPMAALSNRLSIMKRCLWGIELFLFRKQNLHWQNYQKIANYHYFCSRKKRKGTKKIKKERKQAMTHTTL
LLTRHGETVDNVNQIMQGQTQGQLTTHGREQAEVVAKEMADRQIDAFVSSDLKRAIDTCHIIALPHKAPV
VTTPLLRERDWGGFTGRYIPDLKGEPWPDDIETMDDLKARAARFIEFIRHSYPGKTVLARGPRHH-
>BLAST result
Histidine phosphatase family protein [Prevotella sp.] MBR2242956.1
(IdentityL 75%)

8. TS24F1 (Library 40101)
>DNA sequence
ATGCAGACCGCGCTGAACGGCATCTCGGCTTCCACCGACGAGCTGAACACCCTGCTGGACGCATCCACCC
AGATCAAAAACGGCATTGCACAATTGGACGAGGGCGCGGCCCAGCTGGAACAGCAGGTGAGCTTTGACGC
TTACAAAGCCATCCTCAAGGAGAACGGCCTCGACCTTGACGTGGTGAAGGACGGCAATGCCAAAGCCATC
GCCCAGCTGGAACAGCTCTCCAAGCTGATGCCGCAGCTGAAAGATGTCATTCTGCTGCTGCAGGGCTCCA
CCGCGAACATCGACGCGATGCAGACCTACCTCGACACGGTCAACGGAGGCATTGCCCAGCTGCACGAGGG
CAGCAGCACCCTGAACAGCAGCTACGGCGAGTTTGACGCGGGCGTGCAGCAGCTGGCCGGTGTGCTCACC
GGGATGCTGGGCAATCTCTCGGTTCTCACCGACGGCGTGAACCAGCTGGCCAGCCAGTACGCGAAGCTTG
ACGACGGCCTGAACGCCTACACCGGCGGCGTGGCCCCAGTTGAAAGCGGGCGTGGCGCAGCTGGCCGAGG
GTGCGGCCCAGCTGACCGGCGGCACCGGCGAGCTGCGGGANNACGTCTCCGGCATCGACATGGGCGATGA
>Amino acid sequence
MQTALNGISASTDELNTLLDASTQIKNGIAQLDEGAAQLEQQVSFDAYKAILKENGLDLDVVKDGNAKAI
AQLEQLSKLMPQLKDVILLLQGSTANIDAMQTYLDTVNGGIAQLHEGSSTLNSSYGEFDAGVQQLAGVLT
GMLGNLSVLTDGVNQLASQYAKLDDGLNAYTGGVAPVESGRGAAGRGCGPADRRHRRAAGXRLRHRHGR-
>BLAST result
Hypothetical protein [Faecalibacterium prausnitzii] WP_097800489.1
(Identity: 98%)

9. TS30F1 (Library 40203)
>DNA sequence
ATGCCGTTTACAAGCAGGGGAGGCGAAATGAGTGCTGGCAAGGGATTGTTGCTCGTCATCTGCCTGTTAT
TTCTGCCGTTGAAGTCTGCAATGGCGCTGAACTGCTATTTTGGTACATCTGGTGGCGCAGTAGAAAAATC
AGAAGCGATTCAACCGTTTGCTGTACCAGGCAATGCCAAACCCGGTGATAAGATCTGGGAATCTGACGAT
ATTAAAATTCCCGTCTATTGTGACAACAATACCAACGGCAACTTTGAAAGCGAGCATGTCTACGCCTGGG
TAAACCCCTATCCGGGGGTGCAGGATCGTTATTATCAACTCGGCGTGACATACAACGGTGTCGATTACGA
TGCCAGTCTGGGAAAAAGTCGCATCGACACTAACCAGTGTATCGACAGTAAGAACATTGATATTTATACC
CCTGAGCAGATCATCGCGATGGGATGGCAGAACAAAATTTGCTCGGGCGATCCCGCTAATATTCACATGT
CGCGTACCTTTCTCGCTCGCATGCGGTTATACGTCAAAATACGAGAAATGCCGCCGCATGATTATCAAAG
TACGCTTAGCGACTATATCGTCGTGCAATTTGACGGTGCCGGTAGCGTTAACGAAGACCCTACTGCCCAA
AACCTGAAATATCATATTACTGGTCTGGAAAACATTCGCGTGCTGGATTGCAGCGTCAATTTTTCCATTT
CACCGGAAACACAGGTGATTGATTTTGGTAAATTTAATTTGCTGGATATACGTCGCCACACAATGTCGAA

AACGTTCAGTATTAAAACGACCAAGAGCCAAAACGATCAATGCACCGACGGATTTAAAGTCAGTTCCTCC
TTTTATACCGAAGAAACGTTGGTTGAAGAAGATAAAGCATTGTTGATTGGTAATGGTCTGAAGCTACGTT
TACTGGATGAAAACGCCTCGCCTTATACCTTCAATAAATATGCTGAATACGCCGATTTCACCAGTGACAT
GTTGGTCTACGAAAAAACCTATACGGCTGAACTTTCGTCCATTCCAGGCACCCCCATCGAGGCTGGCCCC
TTCGATACAGTGGTGCTTTTTAAGATTAACTATAACTGA

>Amino acid sequence
MPFTSRGGEMSAGKGLLLVICLLFLPLKSAMALNCYFGTSGGAVEKSEAIQPFAVPGNAKPGDKIWESDD
IKIPVYCDNNTNGNFESEHVYAWVNPYPGVQDRYYQLGVTYNGVDYDASLGKSRIDTNQCIDSKNIDIYT
PEQIIAMGWQNKICSGDPANIHMSRTFLARMRLYVKIREMPPHDYQSTLSDYIVVQFDGAGSVNEDPTAQ
NLKYHITGLENIRVLDCSVNFSISPETQVIDFGKFNLLDIRRHTMSKTFSIKTTKSQNDQCTDGFKVSSS
FYTEETLVEEDKALLIGNGLKLRLLDENASPYTFNKYAEYADFTSDMLVYEKTYTAELSSIPGTPIEAGP
FDTVVLFKINYN-

>BLAST result
Hypothetical protein P12B_c0686 [Escherichia coli P12b] AFG39563.1
(Identity: 99%)

10. TS36R1 (Library: 40203)

>DNA sequence
ATGCACAGCATTTATTTTGCCCGCCACGGCGAGACGGTCTGGAACGTGGAGAATAAGATCTGCGGCATGA
CCGACAGCCCCCTCACCGAAAAAGGCCGTCAGCAGGCCCGGGAGCTGGGCCGGAAGGTCAAGGAAAGCGG
CGTCCACATCGACGAGATATTGTACTCGCCCCTGAgCCGCGCCGCCGACACCGCAAAGGCCGTGGCCGAG
GCCACCGGCCTGCCCGCCCGCTGTGAGCCCCGCCTGCGGGAGCAGTGCTTTGGCAAATACGaaGGCACGC
CCCGGGACGGCGCagagTTTCGCATCTCTAAGACCCATTTTGCCGACCGCTACGATGGCGGCGAGAGCAT
GATGCAGCTGGCCCAGCGCATTTATAATCTGTTGGACGAGCTGCGGCAGGATGAGAACAAGACCTATCTT
CTCGTGGCCCACAACGGCATCGCCCGTGTAGTGGAGTCCTATTTCCACGACATGACCAACGAGGAGTATT
CCGCCGCCGGCATCAAAAACTGTGAGTTGGTGGAATACCGGTTCGAATAA

>Amino acid sequence
MHSIYFARHGETVWNVENKICGMTDSPLTEKGRQQARELGRKVKESGVHIDEILYSPLSRAADTAKAVAE
ATGLPARCEPRLREQCFGKYEGTPRDGAEFRISKTHFADRYDGGESMMQLAQRIYNLLDELRQDENKTYL
LVAHNGIARVVESYFHDMTNEEYSAAGIKNCELVEYRFE-

>BLAST result
Histidine phosphatase family protein [Faecalibacterium prausnitzii]
100% WP_158395513.1 (Identity: 100%)
